# Click “Like” to Change Your Behavior: A Mixed Methods Study of College Students’ Exposure to and Engagement With Facebook Content Designed for Weight Loss

**DOI:** 10.2196/jmir.3267

**Published:** 2014-06-24

**Authors:** Gina Merchant, Nadir Weibel, Kevin Patrick, James H Fowler, Greg J Norman, Anjali Gupta, Christina Servetas, Karen Calfas, Ketaki Raste, Laura Pina, Mike Donohue, William G Griswold, Simon Marshall

**Affiliations:** ^1^Center for Wireless and Population Health Systems, The Qualcomm Institute/Calit2Department of Family and Preventive MedicineUniversity of California San DiegoLa Jolla, CAUnited States; ^2^Center for Wireless and Population Health Systems, The Qualcomm Institute/Calit2Department of Computer Science and EngineeringUniversity of California San DiegoLa Jolla, CAUnited States; ^3^Center for Wireless and Population Health Systems, The Qualcomm Institute/Calit2Department of Medicine, School of MedicineUniversity of California San DiegoLa Jolla, CAUnited States; ^4^Center for Wireless and Population Health Systems, The Qualcomm Institute/Calit2University of California San DiegoLa Jolla, CAUnited States

**Keywords:** overweight, obesity, students, social networking, behavior, social behavior

## Abstract

**Background:**

Overweight or obesity is prevalent among college students and many gain weight during this time. Traditional face-to-face weight loss interventions have not worked well in this population. Facebook is an attractive tool for delivering weight loss interventions for college students because of its popularity, potential to deliver strategies found in successful weight loss interventions, and ability to support ongoing adaptation of intervention content.

**Objective:**

The objective of this study was to describe participant exposure to a Facebook page designed to deliver content to overweight/obese college students in a weight loss randomized controlled trial (N=404) and examine participant engagement with behavior change campaigns for weight loss delivered via Facebook.

**Methods:**

The basis of the intervention campaign model were 5 self-regulatory techniques: intention formation, action planning, feedback, goal review, and self-monitoring. Participants were encouraged to engage their existing social network to meet their weight loss goals. A health coach moderated the page and modified content based on usage patterns and user feedback. Quantitative analyses were conducted at the Facebook post- and participant-level of analysis. Participant engagement was quantified by Facebook post type (eg, status update) and interaction (eg, like) and stratified by weight loss campaign (sequenced vs nonsequenced). A subset of participants were interviewed to evaluate the presence of passive online engagement or “lurking.”

**Results:**

The health coach posted 1816 unique messages to the study’s Facebook page over 21 months, averaging 3.45 posts per day (SD 1.96, range 1-13). In all, 72.96% (1325/1816) of the posts were interacted with at least once (eg, liked). Of these, approximately 24.75% (328/1325) had 1-2 interactions, 23.39% (310/1325) had 3-5 interactions, 25.13% (333/1325) had 6-8 interactions, and 41 posts had 20 or more interactions (3.09%, 41/1325). There was significant variability among quantifiable (ie, visible) engagement. Of 199 participants in the final intervention sample, 32 (16.1%) were highly active users and 62 (31.2%) never visibly engaged with the intervention on Facebook. Polls were the most popular type of post followed by photos, with 97.5% (79/81) and 80.3% (386/481) interacted with at least once. Participants visibly engaged less with posts over time (partial *r*=–.33; *P*<.001). Approximately 40% of the participants interviewed (12/29, 41%) reported passively engaging with the Facebook posts by reading but not visibly interacting with them.

**Conclusions:**

Facebook can be used to remotely deliver weight loss intervention content to college students with the help of a health coach who can iteratively tailor content and interact with participants. However, visible engagement with the study’s Facebook page was highly variable and declined over time. Whether the level of observed engagement is meaningful in terms of influencing changes in weight behaviors and outcomes will be evaluated at the completion of the overall study.

## Introduction

Approximately 1 in 3 college students are overweight or obese [1] and most students gain weight during college [2]. Total weight gain over 3 years has been estimated at 10 pounds and is characterized by a significant increase in percent body fat and a decrease in lean muscle mass [3]. Weight gain during this time can initiate a trend toward long-term weight gain [4] and increase ones’ risk for developing Type 2 diabetes [5], coronary heart disease [6-8], and depression [9] in adulthood. Weight gain in young adults is also associated with psychological distress, such as low self-satisfaction and a loss of identity [10].

Commonly used approaches to weight loss may not be effective for this population [11,12]. For example, more than half of the college students enrolled in a study that required just 1 on-campus counseling session per week quit after 3 months with 75% citing lack of time as their reason for leaving [11]. College students may be less likely to drop out of interventions delivered via the Internet (eHealth) [13] or mobile/social media (mHealth) [14] because these interventions can be delivered remotely and conveniently. At least one Internet-based randomized controlled trial (RCT) demonstrated good retention with just 18 of 159 participants lost to follow-up during the intervention [15].

Both mHealth and eHealth interventions have the potential to engage college students because online, social, and mobile tools are already integrated into their lives. The young adult population, age 18-29 years, has the highest smartphone penetration [16] and 88% of college students connect to the Internet using a mobile device [17]. College students are also highly connected via online social networks [17] with Facebook being the most popular [18]. Compared with other Facebook user groups, young adults more frequently access the site each day to update their status, comment, and “like” their friends’ content [19].

In addition to its popularity, Facebook is an attractive platform for health promotion because it fosters interactivity among users and encourages content creation [20]. The Facebook user experience is a combination of human-computer interaction and computer-mediated communication [21]. Therefore, health behavior interventions should consider how participants use Facebook (ie, human-computer interaction) as well as anticipate how participants communicate through Facebook (ie, computer-mediated communication). Facebook mediates communication between friends by facilitating the sharing of personal content and the provision of feedback. Friends virtually interact with one another by posting photos, messages, and links, and by liking and commenting on friends’ posts. This creates a community where social interactions account for much of the time spent on the site. Health interventions can leverage this community by creating a Facebook page to reach participants while they interact in real time. A complete review of Facebook’s features in the context of behavioral research was published by Wilson et al [22].

Similar to being part of an online health community, Facebook users can motivate one another to achieve their goals [23]. However, Facebook users differ from online health community members in terms of motives for site use, strength of social ties on the site, and activities engaged in while on the site. For example, Facebook users are frequently members of many different nonoverlapping friendship networks and, within each, preferences for sharing personal health information may vary [24]. Thus, users are selective about how and what they share on Facebook, balancing the need for impression management and social support [24,25].

Users commonly “lurk” online, passively reviewing content without visibly connecting to other users or information [26]. Lurking behavior can be prompted by a desire for privacy, a function of individuals’ virtual behavior tendencies, or some combination of factors. Benefiting from membership in online social networks, however, may not require visible participation: individuals who passively engage have reported receiving high levels of social support for weight loss [27]. Online interventions encouraging social support may increase engagement [28] and exchanging social support on social networking sites (ie, Twitter) has been linked to weight loss [29].

Facebook enables the provision of timely social support as well as the giving and receiving of behavioral feedback, which is also important for weight loss. Meta-analytic data suggest that interventions that give participants’ feedback on their diet and physical activity are more effective than those that do not (pooled effect size: Cohen’s *d*=0.42) [30] and that delivering personally relevant feedback improves adherence to online interventions (pooled effect size: Cohen’s *d*=0.22) [31]. Qualitatively, individuals have referred to their mobile devices as “virtual companions” that provide real-time support and feedback [32].

Another way that Facebook might be leveraged to change weight loss behaviors is through normative influence. For example, individuals who received messages on Facebook encouraging them to vote in a presidential election that were based on social norms were significantly more likely to vote than those who received an informational message [33]. Moreover, this message spread within individuals’ social networks, increasing the likelihood that the recipients’ friends and friends of friends would vote [33]. Thus, weight loss interventions using Facebook might be able to capitalize on participants’ social networks to promote the spread of healthy dietary and physical activity behaviors [34].

Although Facebook enables the delivery of evidence-based behavior change techniques, such as the provision of social support and positive normative influence, deploying interventions based on traditional theoretical frameworks via this medium may not work. Facebook interactions are intermittent (asynchronous), involve multiple actors, and social norms dictate observable communication among users. As a result, interventions delivered through Facebook are inherently dynamic and fluid, requiring an adaptable theoretical approach.

An alternative to using traditional theories to design online intervention content is to use a behavior change technique framework, such as the one formulated by Michie and colleagues [35,36]. Behavior change techniques represent the smallest identifiable components of an intervention and can be used flexibly, which is ideal for a technology-based intervention [37]. For example, multimedia learning highlights the importance of “scaffolding,” or adjusting the degree of difficulty of the presentation of information based on the learner’s status (ie, novice to expert). During the course of an intervention, participants develop mastery and messages delivered at the outset may no longer prove useful [37]. In addition, behavior change techniques support specific instructions on how to prompt behavior change, facilitating their adaptation to technology-based interventions [38]. Also, the specificity of behavior change techniques makes clear the competencies required to deliver the technique. For example, modeling/demonstrating the behavior is a behavior change technique that is more difficult to deliver remotely than prompting action planning, which involves detailing when, where, and how often a behavior will be performed [36].

To our knowledge, no published studies have used a behavior change technique framework to design and deliver intervention content via Facebook. However, Michie and colleagues’ framework has been used to analyze the content of Internet-based interventions [39] and has been incorporated into instruments used to analyze the theoretical content of mobile apps [40] and online intervention adherence [31]. This framework has also been used to conduct a meta-regression of 129 diet and physical activity interventions, which found that interventions using self-monitoring plus at least one other self-regulatory technique from control theory [41] were the most effective at changing behavior (Cohen’s *d*=0.42) [30]. A recent review found that the most effective Internet-based interventions used the most behavior change techniques [39], suggesting that delivering multiple behavior change techniques through Facebook may be a valuable approach to improving health behaviors.

In addition to developing an appropriate intervention framework, it is equally important to consider how using Facebook will impact the acceptability of and adherence to the intervention. The few studies published to date suggest that young adults accept behavioral interventions that use Facebook. For example, Cavallo et al [42] tested the feasibility of a Facebook-based social support intervention to increase physical activity among sedentary college students and found that two-thirds of participants would recommend the program to friends. However, they did not find the Facebook condition to be more effective than the control condition in increasing students’ perceived social support for physical activity or self-reported physical activity [42]. Similarly, Napolitano et al [43] demonstrated that a Facebook intervention for weight loss was popular among college students, but did not find Facebook intervention elements alone to be effective. However, neither of these studies fully leveraged Facebook’s capacity to serve as a dynamic 2-way communication channel between the participants and the study, and between participants and their existing friendship network.

Whether social networking sites can be used as a novel setting for health promotion is currently a matter of debate with some concluding that the dearth of evidence linking online participation with offline health activities and/or positive health outcomes suggests investigators should proceed with caution [20]. What is largely missing from this conversation, however, is the recognition that standardized and validated metrics for evaluating intervention exposure and engagement are required before Facebook intervention efficacy can be addressed.

Evaluating exposure and engagement requires accurately capturing metrics of intervention delivery and adherence, yet most early Facebook research has either tallied these data manually [42,43] or used publically available Facebook Insights data [44]. Manually entered data are prone to human error and unlikely to be comprehensive. Facebook Insights data are limited because they are aggregated up to the page level and individual participants’ data cannot be visualized. In addition, all fans of the page are included in Insights’ capture, which contaminates analyses of open pages because nonstudy participants may be responsible for some/most of the page interactions. Also, Insights’ data can have questionable internal validity. For example, Insights’ data defines *reach* as the message being visible on one’s personal page (ie, news feed) despite the fact that there is no evidence that the individual has attended to this information. An alternative to objectively capturing engagement and exposure data is to ask participants to self-report their Facebook use during the course of an intervention. Participants in at least 1 Facebook-based intervention self-reported on the frequency with which they saw the study’s posts in their newsfeed, how often they visited the study page, and other metrics [45]. These self-report data may be particularly useful for capturing data on lurking [26].

Also prior to assessing Facebook intervention efficacy, it is important to consider what defines a satisfactory level of exposure and engagement. Paradata (eg, number of website log-ins) have been used to explore participant involvement and identify the more/less popular components of online interventions [46]. However, extending this type of data capture to online social networking sites in the context of a weight loss RCT has not been reported.

The present study had 2 goals. First, to present a way to objectively quantify and qualitatively explore participant exposure to and engagement with the Facebook page that is currently being used in a weight loss RCT of 404 overweight/obese college students. Second, to describe participant exposure to and engagement with various weight loss campaigns grounded in a behavior change technique model and delivered through Facebook. This study defined overall exposure as the number of Facebook posts delivered by the health coach (ie, dose delivered) and engagement as the number of posts participants interacted with (ie, dose received) as well as participant-initiated posts. These data are based on an interim analysis of a 2-year RCT evaluating a weight loss program for young adults [47].

## Methods

### Participants

The study used a rolling recruitment strategy whereby participants were recruited over a year’s time. From May 2011 to May 2012, 404 students were recruited from 3 Southern California universities to participate in a weight loss intervention delivered through social and mobile technologies called project SMART (social and mobile approach to reduce weight). SMART is 1 of 7 studies funded by the National Heart, Lung, and Blood Institute to target weight loss/weight control in young adults. The primary outcome of SMART is weight loss at 24 months from baseline.

Inclusion criteria were: (1) age 18 to 35 years, (2) body mass index (BMI) 25-40 kg/m^2^, (3) owns a personal computer, (4) owns a mobile phone and uses text messaging, and (5) a Facebook user or willing to join. Exclusion criteria were: (1) comorbidities of obesity that require clinical referral (eg, diabetes), (2) psychiatric or medical conditions that could prohibit study compliance (eg, bipolar disorder), (3) taking weight-altering medications, (4) pregnant or intending to get pregnant over the next 2 years, and (5) enrolled in or planning to enroll in another weight loss program.

### Procedures

Online and in-person recruitment strategies included banner ads on campus websites, a Facebook page, listservs, health fairs, and student orientations. These recruitment channels directed potential participants to the study website where they could take an eligibility survey and learn more about the study. Eligible participants were randomized into 1 of 2 groups: social and mobile intervention (n=202) or online education-only control (n=202). All participants provided written informed consent and the university’s Institutional Review Board approved the study protocol (University of California San Diego, California State University San Marcos, and San Diego State University).

Participants attended a measurement visit at baseline and every 6 months for 24 months, conducted at the students’ university. Self-report data collected at the visit included demographics, dietary and physical activity habits, psychological symptoms/states, quality of life, Facebook usage, and information about participants’ social networks. University health center staff also measured participants’ height, weight, waist circumference, and blood pressure at every visit. Participant compensation increased by US $5 after each completed visit, from US $20 at baseline to US $50 at 24 months. Participants who completed their visit within a 2-week window of their scheduled visit (1 week prior to 1 week after) received double the compensation (eg, US $100 for their final visit at 24 months).

### Intervention and Control Conditions

The intervention group had access to a study-specific website, blog, apps, Facebook page, text-messaging component, and a health coach. Upon entering the study, all intervention participants were asked to like the Facebook page. After liking the page, users were considered fans of the page and could see all posts in their news feed. Because the Facebook page was open, nonstudy participants could also become fans and view and engage with its content.

A health coach (a registered dietitian) remotely delivered all intervention content to the 202 intervention participants. The health coach moderated the Facebook page, and posted to the blog. Participants could contact the health coach up to 10 times (Lifelines) via Skype, email, phone, or text. Alternatively, the health coach could reach out to participants a maximum of 10 times (Lifesavers). The health coach used Lifesavers when participants gained >5 pounds since study entry or had not logged into at least 1 of the study’s tools in >1 month.

SMART intervention tools were branded as ThreeTwoMe, symbolizing the role of the college student within a broader social ecological context [48,49]. *Three* represents the student in his/her community, *Two* represents the student in his/her friendship network, and *Me* represents the student’s individual behavior change needs. All intervention tools used the same ThreeTwoMe brand identity (ie, logo, imagery, colors). The control group received a self-guided educational program called SMART Health Tools that offered standard-of-care information disseminated via a different study website with minimal interactive features. Online log-ins were monitored weekly to detect possible contamination of control group participants (ie, exposure to the intervention). Additional details about the intervention’s methods have been published elsewhere [47].

### Facebook

#### Conceptual Model

**Figure 1:**
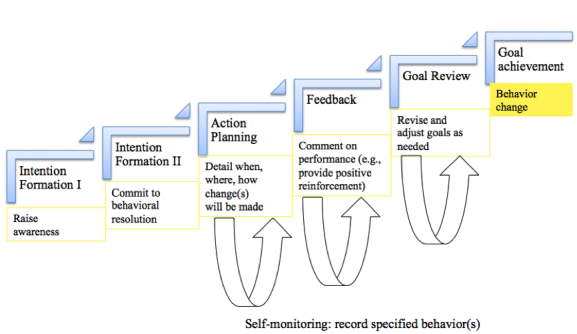
Conceptual model iSIMPLE (Intention formation, Self-monitor, Make plans, Execute) used in the design of the SMART study’s Facebook campaigns for weight loss behaviors.

Abraham and Michie’s 26-item behavior change taxonomy [35] was used to create a conceptual model called iSIMPLE (intention formation, self-monitor, make plans, execute) for the Facebook campaigns (see Figure 1). The taxonomy is grounded in theory known to enhance behavior change, including the theory of reasoned action [50], social cognitive theory [51], control theory [41], operant conditioning [52], theories of social comparison [53], and theories of social support [54]. iSIMPLE was based on the 5 most effective behavior change techniques identified in a meta-regression analysis [30]. These techniques are intention formation, action planning, feedback, goal review, and self-monitoring.

#### Facebook Campaigns

Campaigns were intervention periods (eg, week of Thanksgiving) with a specific relevant theme (eg, mindless eating). iSIMPLE was used to design Facebook campaigns that were either sequenced or nonsequenced. Sequenced campaigns implemented the behavior change techniques in a hierarchical manner, consistent with a temporal sequence in how behavior change is thought to occur [55,56]. For example, participants were first asked to join the campaign and pledge to change their behavior (ie, intention formation). Pledge requests were followed with action planning wherein participants were prompted to describe when, where, and how they would change their behavior. Throughout the campaign, the health coach provided feedback and conducted goal reviews, prompting participants to revise and adjust their goals as needed. While participants worked toward behavior change, the health coach also prompted for self-monitoring. Participants were prompted to observe and record their diet and physical activity behaviors on Facebook (eg, Facebook poll) as well as on the study website and apps. For example, participants were asked to record their daily steps during a pedometer campaign by posting their steps on Facebook (public option) or via text message (private option).

Nonsequenced campaigns used the same behavior change techniques, but participants were not explicitly asked to join the campaign and messages pushed to participants were not systematically ordered. Nonsequenced campaigns exposed participants to similar content as sequenced campaigns, but the posts did not outline an explicit campaign-based goal the participant should work toward. For example, an unstructured January campaign delivered motivational posts that mapped onto various behavior change techniques, but participants were not asked to complete specific tasks to meet a campaign goal.

Designing and delivering sequenced and nonsequenced campaigns enables the comparison of ordered behavior change techniques. It was theorized that having 2 different campaign structures may maximize overall participant engagement. Some users may be more interested in being challenged and pledging to participate in a campaign, whereas others may prefer to receive content that asks less of them in terms of overt online participation. It also may be the case that delivering too much dynamic content is problematic: there is some evidence that adding layers of complexity to online interventions can decrease participant engagement and intervention effectiveness [57], exacerbating the problem of reduced motivation to engage with online tools over time [31].

Behavior change techniques not specified in iSIMPLE were also used as needed. For example, the ThreeTwoMe health coach could provide contingent rewards (ie, prizes based on completion of an activity) and model behavior (eg, post a link on how to correctly do a push-up). The health coach also delivered behavioral cues to action messages [58,59] alongside/in place of preplanned messages (ie, Facebook posts that were designed a priori as part of a campaign) dependent upon user engagement and participant feedback. For example, the message “30 minutes of exercise per day is recommended, even if it’s split apart. That’s only 3% of your day, so start moving!” was sent as a behavioral cue message in the late morning after students’ gave feedback that this was a good time to remind them to plan for their day. The health coach also delivered “filler” messages that included posts tailored to our audience, such as “Good luck with finals this week!”

Although the iSIMPLE model is focused on the individual, it is supported by the social support platform inherent to Facebook’s architecture. Some of the Facebook campaigns asked participants to engage their social networks to make successful behavior change more likely. For example, Facebook posts often prompted participants to be active with their friends and discuss their goals with them. In-between campaigns, Facebook content was often less theoretically informed and did not necessarily map onto behavior change techniques. For example, a post to promote eating vegetables was an infographic of the actor Patrick Dempsey holding a bushel of kale.

### Process Measures and Analysis Plan

The findings presented here represent a way in which exposure to and engagement with Facebook can be conceptualized for the purposes of health behavior intervention research. These analyses focused on Facebook posts delivered by the health coach and posts received by study participants. The measures used to define exposure and engagement are presented subsequently.

The study’s Facebook page was created on August 7, 2011, the same day the first participant was randomized. General posts aimed at helping participants lose weight and be healthy were delivered for the first ~45 weeks of the intervention. During this time, the research team focused on integrating the various channels (eg, apps, text messaging, Facebook) and testing various Facebook messages. The first campaign was launched on June 21, 2012.

For purposes of the present study, Facebook data from August 7, 2011 and May 27, 2013 were collected. These dates include the first (August 7, 2011) and last day (May 27, 2012) of participant randomization. It is important to point out that because of rolling recruitment, participants were exposed to varying amounts of Facebook content delivered before the start of the first campaign. For example, participants randomized in the first month of recruitment were exposed to approximately 10 months of noncampaign content, whereas participants randomized in the last month of recruitment were exposed to approximately 2 months of noncampaign content. However, all participants were exposed to the same 8 campaigns analyzed here as well as the same noncampaign content posted in-between campaigns (ie, noncampaign content delivered after the start of the first campaign).

For participant-level analyses, the first 12 months of data based on each participant’s start date were used. For post-level analyses, all 21 months of data were used so that the first 8 campaigns could be evaluated.

Facebook query language was used to retrieve data from Facebook’s social graph. Data were downloaded in JavaScript Object Notation (JSON) format. Given that this study’s Facebook page was open, nonstudy participants could like it and become a fan. Therefore, the exported data contained some nonparticipant Facebook activity. However, these data were excluded by only including data associated with Facebook identification numbers belonging to study participants. The data obtained via Facebook’s social graph were merged with baseline survey data in SPSS version 20 (IBM Corp, Armonk, NY, USA).

### Exposure

#### Dose Delivered

*Dose delivered* was defined at the Facebook post-level unit of analysis as the number of unique posts made by the health coach to the study’s Facebook page. The number of health coach posts was summed over the course of the study’s first 21 months (August 7, 2011-May 27, 2013). Therefore, this metric includes posts made both during and outside of campaigns. An example of a status update made by the health coach outside of a campaign was: “This is a great checklist for a healthy pantry. Are you in good shape??” This post included a link to an article about tips for keeping a healthy pantry. Campaign versus noncampaign posts were not always qualitatively different when considered individually; rather, posts made during campaigns were qualitatively different in aggregate (ie, because they were part of a cohesive message/plan).

#### Feedback Delivered

The number of comments the health coach made on participant posts and the number of times the health coach liked participant posts were summed and used as a measure of the dose of feedback delivered.

### Engagement

#### Dose Received

Dose received was defined at the post-level and the participant-level unit of analysis. *Post-level dose received* was defined as the number of posts made by the ThreeTwoMe health coach that participants interacted with. Participants could interact with a post by liking, commenting, sharing, or answering (if the post was a poll). Post-level dose received was analyzed as a binary variable (post was interacted with: yes or no) as well as a continuous variable (total number of interactions with the post). *Participant-level dose received* was defined as the number of participant interactions stratified by interaction type (eg, like). For example, a participant who liked a photo on the ThreeTwoMe page was said to have “received” that post.

#### Participant-Initiated Posts

The number of posts participants made to the study’s Facebook page was summed. These data represent participant activity that was either motivated by the participant independent of the health coach prompting for it or the health coach calling for participants to post. For example, a participant may have heard about a diet from a friend and sought the health coach’s advice by posting about it. Differently, a participant may have posted a picture of a meal he/she recently made that met a challenge the health coach put to participants. Both types of participant-driven communications made directly on the ThreeTwoMe page may represent a higher level of engagement than dose received because they involve participant-initiated contact with the study as opposed to study-initiated contact with participants. For example, a participant who proactively visited the ThreeTwoMe page and posted a message about how many steps he/she took that day may be more engaged than a participant who only liked messages posted by the health coach that show up in his/her news feed.

Facebook posts can extend into a long thread of comments and corresponding likes, representing interactivity between participants and the health coach and/or participants and participants. For the purposes of this study, only interactions between participants and the health coach were considered. *Interactions* were defined as participant comments, etc, made to posts originating from the health coach (dose received) and likes or comments from the health coach to participants’ posts (feedback delivered). Participant comments, etc, that were made to participant-initiated posts were not considered because the focus was on dose received from the health coach, not dose received from fellow participants. Table 1 summarizes the aforementioned definitions of exposure and engagement.

Participants who interacted with the ThreeTwoMe Facebook page at least once per week were categorized as highly active and those who interacted at least twice per month were categorized as active. The remaining participants were categorized as either somewhat active (interacts >once per month) or minimally active (interacts <once per month). These mutually exclusive cut points were based upon Facebook user patterns seen in this intervention, but are similar to categorizations delineated in earlier work. For example, a previous study categorized participants as active if they posted to Twitter at least once per week [29].

**Table 1:** Defining measures of intervention exposure and engagement on Facebook.

Intervention		Definition	Types of posts/interactions	Direction of posts/interactions
**Exposure**				
	Dose delivered	Posts made by the health coach on the ThreeTwoMe Facebook page	Posts: status update, photo, link, poll,^a^ video	Health coach to ThreeTwoMe page
	Feedback delivered	Health coach interactions with posts made by participants on the ThreeTwoMe Faceook page; also can be health coach interactions with comments made by participants in response to ThreeTwoMe posts	Interactions: like, comment	Health coach to participant
**Engagement**				
	Dose received^b^	Participant interactions with ThreeTwoMe Facebook posts delivered by the health coach	Interactions: like, comment, poll response, share	Participant to ThreeTwoMe page
	Participant-initiated posts	Posts made by participants on the ThreeTwoMe Facebook page	Posts: post, photo, video, link	Participant to ThreeTwoMe page

^a^Facebook removed the poll feature in April 2013.

^b^Dose received was analyzed at the post-level (post interacted with: yes/no) and at the participant-level (number of participant interactions stratified by interaction type).

#### Semistructured Interviews

A total of 29 participants (15 treatment, 14 control) were interviewed between May and July of 2013 as part of a qualitative study. Participants were “intercepted” at the end of one of their measurement visits and those who agreed to be interviewed were provided with an additional US $25 incentive (gift card to Target). Intercepted participants comprise a convenience sample; however, a concerted effort was made to sample from males and females, and students from all 3 universities. Although participants from all 3 universities were approached, most of the sample came from the University of California San Diego (23/29, 80%). Most participants interviewed were female (17/29, 59%). Interviews lasted between 30 and 60 minutes. Interviewees were asked about how they used the study’s tools and how they engaged in their social network to meet their health-related goals. All interviews were tape recorded and transcribed. Two investigators independently reviewed the interviews using principles from grounded theory [60].

To explore the common practice of lurking in online health communities [26], selective results from participants’ semistructured interviews are described here. Approximately 40% (12/29, 41%) of participants interviewed described lurking on Facebook. *Lurking* is defined as members of online health communities passively receiving social support and information by reading messages without visibly interacting [23,26,27,61]. On Facebook, lurking can be defined as users viewing posts but not interacting with them in a way that is visible to their social network. For example, commenting on a post makes the post visible to other members of the network and the content and source of the post becomes public, whereas reading or clicking on a post does not make it public. Previous work has found that approximately half of online health community members interact passively and that this type of engagement may play an important role in weight loss efforts [23,27].

## Results

### Overview

Table 2 provides descriptive statistics for the intervention group stratified by gender. A total of 3 of the 202 participants randomized to the intervention group were missing data (final N=199).

At baseline, 16.6% (33/199) of participants self-reported logging onto Facebook zero times per day on either a laptop, desktop, or mobile device, followed by 39.0% (77/199) logging on 1-3 times, 27.6% (55/199) logging on 4-8 times, and 17.1% (34/199) logging on 8 or more times per day. Participants reported spending an average of 60 minutes per day on Facebook (median 60) on both week (IQR 90 min/day) and weekend days (IQR 120 min/day). 

**Table 2:** Baseline characteristics of the intervention group by gender (N=199).

Demographics		Total N=199	Men n=59	Women n=140
Age (years), mean (SD)		22.0 (3.8)	23.0 (4.6)	21.6 (3.3)
**Race, n (%)**				
	White	85 (42.7)	22 (37.3)	63 (45.0)
	Black	7 (3.5)	0 (0)	7 (5.0)
	Asian	45 (22.6)	17 (28.8)	28 (20.0)
	Pacific Islander	9 (4.5)	4 (6.8)	5 (3.6)
	American Indian	1 (0.5)	0 (0)	1 (0.7)
	Other	52 (26.1)	16 (27.1)	36 (25.7)
**Ethnicity, n (%)**				
	Hispanic	63 (31.7)	17 (28.8)	46 (32.9)
Undergraduate (yes), n (%)		159 (79.9)	45 (76.3)	114 (81.4)
**Relationship status, n (%)**				
	Single	100 (50.3)	33 (55.9)	77 (55.0)
	Engaged, committed relationship, married	88 (45)	26 (44.1)	62 (44.3)
	Separated, divorced, widowed	0 (0)	0 (0)	0 (0)
**Anthropometrics, mean (SD)**				
	Body mass index (BMI)	28.7 (3.5)	28.5 (4.7)	28.8 (3.0)
	Waist circumference (cm)	87.0 (10.8)	93.1 (7.7)	84.5 (10.9)

### Exposure

#### Dose Delivered

Table 3 presents the dose delivered and received of Facebook data by post type. The ThreeTwoMe health coach posted 1816 unique messages to the study’s Facebook page over the first 21 months of the intervention, averaging 3.45 posts per day (SD 1.96, range 1-13). Overall, 72.96% (1325/1816) of the posts were interacted with at least once (eg, liked). Most posts were status updates followed by photos, links, polls, and videos. Most messages were posted on a weekday (86.01%, 1562/1816).

**Table 3:** Posts delivered by the ThreeTwoMe health coach and posts received by participants where received is defined as the post having been interacted with at least once.

Dose delivered and received	Total^a^	Type of Facebook post				
		Status update	Photo	Link	Poll	Video
Dose delivered, n (% of total)	1816 (100.00)	802 (44.16)	481 (26.49)	400 (22.03)	81 (4.46)	52 (2.86)
Number of posts delivered per day, mean (SD)	3.45 (1.96)	1.92 (1.11)	1.60 (1.02)	1.44 (0.70)	0.20 (0.30)	1.18 (0.58)
Dose received, n (% of total delivered)	1325 (72.96)	605 (75.44)	386 (80.25)	228 (57.00)	79 (97.53)	27 (51.92)

^a^Data represent posts made since the start of the study’s Facebook page to the end of the eighth campaign (August 2, 2011 to May 27, 2013).

#### Feedback Delivered

The ThreeTwoMe health coach provided ongoing feedback to participants by liking and commenting on their posts. Over the course of the first 21 months of the intervention, the health coach made 675 comments and 389 likes to participant-initiated posts and participant comments to ThreeTwoMe posts.

### Engagement

#### Dose Received-Quantitative Results

Table 3 presents the post-level dose received results. Defining dose received as a binary variable (post interacted with the post: yes/no), intervention participants received 72.96% of the posts (1325/1816 posts were interacted with). Participants interacted most with polls (97.5%, 79/81 of polls posted) and photos (80.3%, 386/481) and the least with videos (51.9%, 27/52). Of the 1816 unique posts, there were 8967 interactions for an average of 4.94 (SD 5.37) interactions per post. Only considering posts that were received (n=1325), there was an average of 6.77 (SD 5.21) participant interactions per post (range 1-37). Approximately 25% (328/1325, 24.75%) of the posts received had 1 or 2 interactions, 23.39% (310/1325) had 3 to 5 interactions, and 25.13% (333/1325) had 6 to 8 interactions. A total of 41 posts had 20 or more interactions (3.09%, 41/1325).

Table 4 presents participant-level dose received by interaction type and participant engagement category. There was high variability among participant engagement with the ThreeTwoMe Facebook page with a range of 0-653 total interactions per participant. In all, 62 participants never engaged with the Facebook page (31.2%, 62/199). There were 32 highly active users (16.1%, 32/199). Likes were the most common type of participant interaction with the ThreeTwoMe posts (69.86%, 4385/6277), followed by comments (22.45%, 1409/6277), and poll responses (4.13%, 259/6277). However, polls were more popular than these data indicate given that there were fewer opportunities to engage with polls than other post types (see message-level dose received data presented in Table 3). Although there were more women (n=26) than men (n=6) in the highly active category, women (mean 49.47, SD 99.19) did not interact with the ThreeTwoMe page significantly more than men (mean 35.17, SD 82.03) (*t*_135_=0.77, *P*=.44).

**Table 4:** Participants’ engagement with the ThreeTwoMe Facebook page by type of Facebook activity and participant engagement category of participants who ever engaged with the Facebook page (n=137).

Engagement category^a^	Total interactions	Type of Facebook interaction^b^				
	n=6277	Likes n=4385	Comments n=1409	Poll responses n=259	Shares n=33	Posts to page^c^ n=191
Highly active (n=32), n (%)	5070 (80.77)	3480 (79.36)	1201 (85.24)	196 (75.68)	20 (60.60)	173 (90.57)
Active (n=17), n (%)	573 (9.13)	442 (10.08)	95 (6.74)	20 (7.72)	3 (9.09)	5 (2.62)
Somewhat active (n=15), n (%)	282 (4.49)	204 (4.65)	63 (4.47)	10 (3.86)	3 (9.09)	2 (1.05)
Minimally active (n=73), n (%)	352 (5.61)	259 (5.91)	50 (3.55)	25 (9.65)	7 (21.21)	11 (5.76)

^a^Categories are mutually exclusive. Highly active participants: interact with the ThreeTwoMe Facebook page ≥1/week; active participants: interact with the ThreeTwoMe Facebook page ≥2/month but <1/week; somewhat active participants: interact with the ThreeTwoMe Facebook page ≥1/month but <2/month; minimally active participants: interact with the ThreeTwoMe Facebook page <1/month.

^b^Data represent quantifiable interaction with the study’s Facebook page over the first year of the study based on participants’ start date.

^c^Posts to page are participant-initiated posts and are independent of a post made by the health coach.

**Figure 2:**
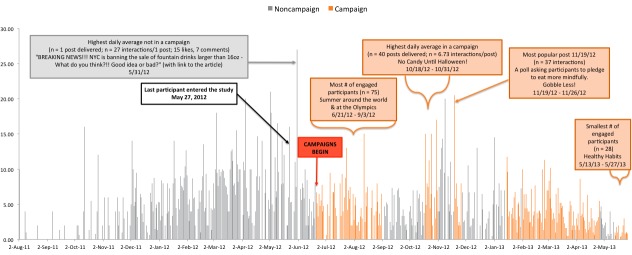
Average daily Facebook interactions per post adjusted for the number of posts delivered.

Figure 2 shows participants’ average number of daily Facebook interactions after adjusting for the number of posts delivered (# of interactions per day/# of posts delivered per day).

**Figure 3:**
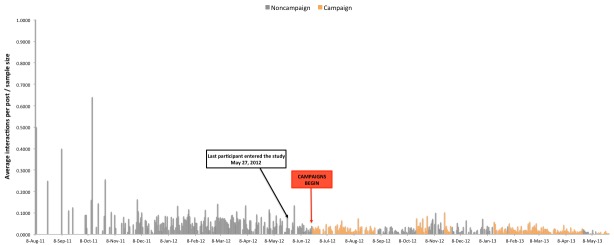
Average daily contribution per person per post delivered.

Figure 3 shows the average contribution per person in terms of daily Facebook interactions after adjusting for the sample size, calculated as (# of interactions per day/# of posts delivered per day)/sample size on that date. The sample size was steadily increasing until May 27, 2012, at which point all 202 participants were in the study. After adjusting for the number of participants in the study on the date the post was delivered, there was a negative correlation between time (date the post was made) and engagement (number of interactions per post) indicating that participants engaged less with ThreeTwoMe posts over time (partial *r*=–.33; *P*<.001).

The decline over time may, in part, be because of decreasing engagement from all but the highly active participants who were responsible for 80.77% (5070/6277) of all Facebook interactions (see Table 4). For example, the trend for declining engagement over time observed in Figure 2 is less pronounced than in Figure 3, which considers the entire sample in the denominator of its engagement metric.

#### Dose Received-Qualitative Results

Two themes emerged concerning participants’ lurking on Facebook. Some participants lurked on Facebook when they interacted with their existing friendship network (ie, it generally characterized their overall social networking behavior), and this extended into their interactions with the study’s page. Others lurked mostly on the study’s page because they did not want to advertise their participation in a weight loss study.

#### “Yeah I Saw It, I Read It”

Participants discussed how they frequently read ThreeTwoMe posts and those made by their Facebook friends without actively engaging. For example, participants said they commonly clicked on links and even if they liked the link, they did not feel obligated to like it on Facebook:

Just because I’m “passive” doesn’t mean I’m ignoring it.

I guess I don’t post a lot on Facebook...I’m more passive...it would be more interesting if [ThreeTwoMe] posted more blogs so then I could easily read or click on [the link].

#### “Facebook is Too Public a Place to Share My Health Information”

A number of those interviewed talked about how they have Facebook friends they are not close with and they don’t feel comfortable sharing with these people. Some participants mentioned taking ThreeTwoMe posts down from their newsfeed once they realized it was visible to their Facebook friends.

...[you] don’t want to share weight loss with people you’ve met like one time in class.

Nobody needs to know I’m in a [weight loss] study.

### Facebook Campaigns

Over the course of the first 21 months of the study, 8 Facebook campaigns were delivered (4 sequenced and 4 nonsequenced). All participants were enrolled in the study by the time the first campaign began. Because of rolling recruitment, however, participants were exposed to the campaigns at different points in their intervention experience. For example, the first campaign began on June 21, 2012, approximately 45 weeks after the first participant entered the study, which meant that some participants were approximately halfway through the 2-year intervention whereas others had just begun the intervention. The last participant to be randomized was less than 1 month into the intervention at the start of the first campaign.

Table 5 presents participant engagement with each of the 8 campaigns. A campaign popularity score that divided the number of participant interactions by the number of unique posts delivered by the health coach was created. The health coach delivered more posts per day (*t*_3.44_=6.13, *P*=.001) over the last 4 campaigns (mean 5.10, SD 0.14) compared to the first 4 campaigns (mean 3.40, SD 0.52).

The length of time participants’ were in the study at the start of a campaign was not significantly associated with the number of Facebook interactions for the same campaign. However, there was a nonstatistically significant difference (*t*_6_=2.08, *P*=.08) between the number of interactions during the first 4 campaigns (mean 4.69, SD 1.39) and the last 4 campaigns (mean 2.72, SD 1.30). Also, the number of unique participants engaged in each campaign declined over time (see Table 5).

**Table 5:** Participant engagement with the ThreeTwoMe Facebook page by Facebook campaign.

Facebook campaigns				Participant engagement,^a^ n		
Popularity rank	Popularity score^b^	Name^c^	Dates	Unique participants engaged	Participant interactions^d^	Unique posts delivered^e^
1	6.73	No candy until Halloween! (S)	10/18/12-10/31/12	51	269	40
2	4.28	Gobble less! (S)	11/19/12-11/26/12	33	107	25
3	4.15	Summer around the world & at the Olympics (NS)	06/21/12-09/3/12	75	893	215
4	4.04	Step it up! Pedometer challenge (S)	02/01/13-02/28/13	51	558	138
5	3.60	Motivation (NS)	01/15/13-01/30/13	53	234	65
6	3.44	Eat right your way (NS)	03/01/13-03/30/13	53	495	144
7	2.27	Get fit anywhere! (S)	04/01/13-04/26/13	43	298	131
8	1.11	Healthy habits (NS)	05/13/13 -05/27/13	28	77	69

^a^Data represent all Facebook interactions during campaigns since the start of the study’s Facebook page to the end of the eighth campaign (August 2, 2011 to May 27, 2013); total number of interactions with the ThreeTwoMe page outside of campaigns was 4965.

^b^Popularity score = # participant interactions / # unique posts delivered.

^c^S: sequenced; NS: nonsequenced.

^d^Includes all likes, comments, shares, and poll answers made in response to ThreeTwoMe posts as well as participant-initiated posts (ie, “posts to page”). Posts to page were adjusted for campaign duration and were included in this total because most posts to page were made in response to campaign requests.

^e^Unique posts delivered are consistently smaller than Participant interactions because the same post could have been interacted with more than 1 time.

**Figure 4:**
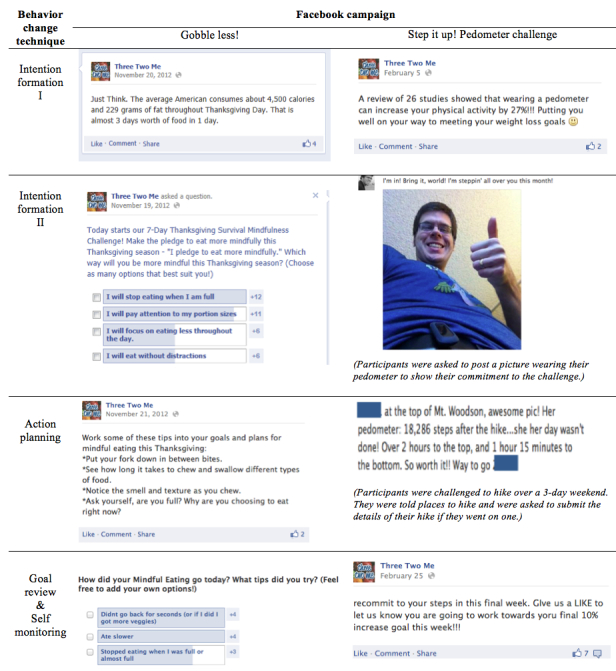
Sequenced Facebook campaign content: Behavior change techniques delivered through Facebook posts.

**Figure 5:**
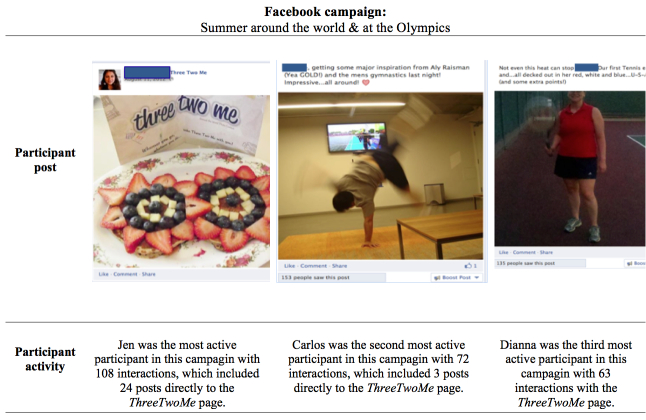
Nonsequenced Facebook campaign content: Participant-inititated posts.

Figure 4 displays sequenced Facebook campaign content stratified by behavior change technique. The health coach gave feedback in the form of likes and tailored responses to participants’ posts. Goal review was frequently tailored to the individual. General goal review posts asked participants to revisit their goals and frequently included a self-monitoring element. Figure 5 shows nonsequenced campaign content and highlights participant-initiated posts. The health coach occasionally posted participants’ content for them because (1) they were either unable to post directly to the page (due to changes Facebook made to their settings) or (2) participants did not want the post to be visible to their Facebook network (ie, due to privacy concerns). When this occurred, these data were counted as part of dose delivered. Note that for Figures 4 and 5 participants consented to having their images used in reports about the study and that names have been changed.

Table 6 presents sequenced and nonsequenced campaigns, describing their aims and the amount of visible participation each received.

**Table 6:** Campaign details for 2 sequenced and 1 nonsequenced campaigns.

Name^a^	Description	Pledging	Dose received^b^
Gobble less! (S)	The campaign focused on mindful eating, encouraging participants to be more aware of what, how much, and why they were eating. Tips for how to eat more mindfully included taking eating breaks by talking with friends and family at the table, waiting until swallowing before taking a second bite, and putting the fork down between bites.	10.6% (21/199) publically pledged to participate by responding to a poll on the ThreeTwoMe Facebook page	76.0% (19/25)
Step it up! Pedometer challenge (S)	Participants were prompted to wear their pedometer to learn their baseline daily steps and encouraged to reach 10,000 steps per day by the end of the campaign by increasing their steps by 10% each week. Participants were given tables with precalculated weekly step increases. The health coach posed 3 mini-challenges during the campaign, aimed at helping participants’ action plan and find fun and creative ways to increase their daily steps.	9.0% (18/199) publically pledged to participate by posting a picture with their pedometer and/or by ‘liking’ a reminder post to take part in the campaign	71.7% (99/138)
Summer around the world & At the Olympics^c^ (NS)	The campaign focused on active travel and had a virtual scavenger hunt whereby participants earned points for completing various challenges, such as being active alone or with friends, and cooking healthy meals. Participants submitted photos or videos as proof they completed the challenge. Another campaign theme was “Take ThreeTwoMe with you all summer long!” whereby participants were asked to take pictures with a ThreeTwoMe postcard while being physically active or eating well.	There was no pledging because this was a nonsequenced campaign	75.8% (163/215)

^a^S: sequenced; NS: nonsequenced.

^b^Dose received is percent of posts participant(s) interacted with ≥1 time (ie, liked, commented, or shared) relative to number of posts delivered for the campaign=(# of posts interacted with/# of posts delivered)*100%.

^c^This was the study’s first campaign.

## Discussion

### Principal Findings

This study suggests that Facebook is a promising medium to deliver theory-based weight loss content to college students. Approximately 73% (1325/1816, 72.96%) of the Facebook posts delivered were liked at least once by 68.8% (137/199) of the intervention participants. Although most of the intervention participants visibly engaged with the study’s Facebook page at least once, interaction frequency was variable, including some participants who never visibly engaged, and engagement diminished over time. Our qualitative results indicate that many participants passively engaged with the page. To our knowledge, this is the first study to present Facebook process evaluation data that is stratified by post type (eg, status update, photo) as well as post interaction (eg, like, comment). These results characterize exposure to and engagement with Facebook in the context of a weight loss intervention. This characterization could be extended to define evaluation metrics in other social network-based behavioral interventions.

### Comparison With Prior Work

Despite interest in Facebook as a setting for health promotion interventions [19,34], little work has been published about how to best capture online metrics of engagement. Although the difficulty in maintaining engagement in online interventions over time is known, conclusions about a study’s efficacy are often made prematurely without first considering usage patterns and nonusage attrition [62,63]. Similar to a sexual health promotion intervention that used Facebook, this study highlights the importance of defining engagement as well as developing a process evaluation plan of how it is to be measured [64]. For example, the results presented here emphasize the importance of measuring visible and nonvisible user engagement: without capturing the common practice of lurking online [26], estimates of engagement will be underestimated.

Although defining an evaluation framework enables the assessment of intervention efficacy, questions regarding the internal validity of engagement metrics remain. Interventions using social networking sites for health promotion are inherently limited by overarching social norms that govern users’ visible behavior on the site [20]. For example, an individual may feel compelled to like a post because it has been liked by many others in the network (ie, social sanctioning) [20]. In addition, it remains unclear whether the like feature is an appropriate measure of engagement or if only comments should be considered as they suggest a deeper level of attention and cognitive processing [64,65]. There is evidence indicating that social network site use should not be considered homogenous; direction of interactions and which features are used differentially impacts outcomes (eg, social capital) [66]. Similarly, different motives for using Facebook predict the use of different Facebook features (eg, the like button) [67] and post type likely influences what features are used.

This study suggests that Facebook engagement metrics should not be lumped together into a single variable because this may obfuscate the interactions that are most relevant in terms of health behavior change. Similarly, metrics of exposure should be stratified so that differences in engagement by post type can be evaluated. Uncovering the most relevant feature use and post type will inform the development of better intervention frameworks.

The intervention framework analyzed in the present study differs from earlier work using Facebook in 3 ways: (1) the use of a behavior change technique framework to generate intervention content and modify content based on usage patterns and user feedback, (2) the amount of engagement and social support provided by the health coach, and (3) the amount of observed engagement with the study’s Facebook page.

The iSIMPLE conceptual model used in SMART was based on behavior change techniques [35] previously demonstrated to be effective in changing weight loss behaviors [30]. Although earlier work has used theory to design message content, a behavior change technique framework has not been used to design and deliver Facebook content. Many online interventions use theory only superficially and intervention techniques are not explicitly mapped onto theory-relevant constructs [39]. Moreover, traditional health behavior theories may be underutilized in online designs because they are not flexible enough to be used in dynamic interventions [68].

In this study, intervention content was supplemented with cues to action or behavioral trigger messages [58] designed in response to participant interaction on Facebook. Delivering messages that are designed based on user feedback and/or user characteristics may be especially important for engagement with online interventions given the prominent role the user plays in computer-mediated interaction [69].

A novel aspect of this study is that the health coach delivered social support by liking and commenting on individual participants’ Facebook interactions. Although Cavallo and colleagues’ Facebook-based intervention had a site moderator who engaged with participants on Facebook, the moderator did not provide direct support to individual participants [42]. Previous work has shown that providing frequent and individualized support motivates overweight and obese individuals [32], and professional support from a health coach can have a significant impact on weight loss [23,70].

In addition to professional support, overweight/obese individuals’ benefit from peer support [71], and peer support may be especially important for college students trying to lose weight [72]. Earlier work using Facebook has promoted the exchange of social support among participants on the study’s Facebook page [42,45], an ad hoc network [34], but has not specifically involved participants’ existing social networks [43].

Ad hoc networks can help individuals lose weight by providing an online space where individuals with shared goals can motivate one another [23], but some are hesitant to share with strangers. Indeed, a sizable number of participants did not visibly engage with the ThreeTwoMe Facebook page. Rather, visible engagement was high among a subset of participants and the qualitative data suggest that a number of participants interacted with the study’s page passively. The proportion of participants who visibly engaged with the study’s page was low but higher than has been reported in other studies. Cavallo et al [42] found that just 45% of participants interacted with the study’s Facebook page once or more over the course of a 12-week intervention, and Napolitano et al [43] found that less than 25% of participants engaged with the study’s Facebook page during an 8-week intervention.

### Limitations

Even though participants in the present study were more engaged compared with earlier work, it remains unknown whether Facebook is a useful medium to deliver behavior change interventions. The extent to which engagement with online interventions equates with behavior change and the mechanisms through which engagement changes behavior are not well understood [46,57,69,73]. Ritterband and colleagues’ model for Internet interventions describes how behavior change is dependent upon user characteristics and the Web application with which the user interfaces [69]. Although this model was developed for more traditional top-down Internet interventions rather than social network-inspired designs, it provides a framework through which limitations of the present study can be considered. Four of the 8 areas deemed critical to intervention effectiveness are discussed in turn.

*Participation* is defined as the application’s ability to interact with the user [69]. Although participants can interact with posts long after they have been posted by scrolling through their newsfeed, there is potentially an overwhelming amount of content to sort through (depending on the number of friends the user has) and posts can become more cumbersome to access over time. For example, when a participant does not actively engage with ThreeTwoMe posts, Facebook’s content-personalization algorithm decreases the frequency of displaying the page’s posts on his/her newsfeed—the less the user interacts with the page the less it appears on their newsfeed. In addition, participants may interact with content passively (ie, lurk) and their engagement is not captured with objectively derived quantitative metrics. Asking participants to self-report their interactions with a Facebook page can circumvent this issue and provide quantitative data on lurking behavior [45]. However, when self-report data are not captured, such as in the present study, quantitative estimates of user engagement may be underestimated.

*Burden* is defined as the ease with which the user can interact with the application [69]. Facebook is widely used [18] and may be especially easy to navigate for college students, but it may become burdensome when leveraged as a behavior change tool part of a multiyear intervention. A recent meta-analysis [31] looking at online interventions found that intervention duration is negatively associated with study adherence and intervention adherence (ie, nonusage attrition) [62], and users report disengaging from Facebook because of boredom, lack of time, or general disinterest with the site [74].

Lastly, engagement and subsequent behavior change are a function of *delivery* and *content* [69]. Although being able to use a variety of types of posts (eg, polls, photos) facilitates the delivery of diverse content, it is unclear which post(s) are most effective. For example, graphic content, such as photos, may be popular but their ability to stimulate behavior change is unknown. In addition, although the present study used an evidence-based conceptual model to design Facebook content, it is unknown the extent to which the posts mapped onto the behavior change techniques as intended. It is also unknown whether delivering more- versus less-structured Facebook campaigns has a measureable impact on behavior change. The more-structured (sequenced) campaigns had a higher average popularity score (4.33) than the less-structured (nonsequenced) campaigns (3.08), but this may be because of seasonal affects given that 2 of the 4 sequenced campaigns coincided with holidays, and/or because of the number of campaigns exposed to given that 2 of the last 3 campaigns delivered were nonsequenced.

Preliminary results from the present study indicate that participant engagement with the Facebook page declined over time. Future work will further evaluate change in engagement over time as well as qualitatively analyze the content of the Facebook interactions observed here. Analyzing the valence and words used in popular posts will inform the creation of better messages with the aim of increasing viral spread. “Going viral” is commonly mentioned as a main draw of using social networking sites for health promotion whereby messages spread among social networks, reaching a vast audience [75].

### Conclusions

Facebook can be used to deliver evidence-based weight loss intervention content designed for college students, but visible participant engagement is low. Benefits of using Facebook include the ability to iteratively tailor content and deliver timely social support and feedback to participants. Participants can engage their existing social networks as well as interact with the study network and the virtual health coach. Facebook also enables the asynchronous delivery of behavior change techniques and participants can view intervention content at their leisure, which may be especially important for RCTs among college students. Future research will examine whether Facebook campaigns based on theory-driven behavior change techniques improve behavioral outcomes and weight loss.

## Abbreviations

iSIMPLE: intention formation, self-monitor, make plans, execute
JSON: JavaScript Object Notation
RCT: randomized controlled trial
SMART: social and mobile approach to reduce weight
